# Development and validation of the Efficacy Safety Score (ESS), a novel tool for postoperative patient management

**DOI:** 10.1186/s12871-017-0344-0

**Published:** 2017-03-28

**Authors:** Erlend Skraastad, Johan Ræder, Vegard Dahl, Lars J. Bjertnæs, Vladimir Kuklin

**Affiliations:** 1 Institute of Clinical Medicine, Medical Faculty, University of Oslo, Oslo, Norway; 2 Department of Anaesthesia and Intensive Care Medicine, Kongsberg Hospital, Vestre Viken HF, Drammensvn 4, 3612 Kongsberg, Norway; 3 Department of Anaesthesia and Intensive Care Medicine, Oslo University Hospital, Oslo, Norway; 4 Department of Anaesthesia and Intensive Care Medicine, Akershus University Hospital, Sykehusveien 25, 1478 Lørenskog, Norway; 5 Anaesthesia and Critical Care Research Group, Department of Clinical Medicine, University of Tromsø, 9037 Tromsø, Norway

**Keywords:** Postoperative care, Postoperative pain, Postoperative nausea and vomiting, Call-out algorithm, Checklist

## Abstract

**Background:**

Several reports have shown that postoperative monitoring of general safety and quality issues, including pain treatment, after discharge from recovery is often non-systematic and inadequate. We suggest a new score with assessment of key recovery parameters, as a supportive tool for postoperative care and a call-out algorithm for need of extra help. The aim of this investigation was to validate the score.

**Methods:**

After suggesting a prototype score from a pilot study in 182 postoperative patients, we performed a Delphi process by using international experts to create consensus on the final score contents and called the revised tool the Efficacy Safety Score (ESS). Then, we performed a prospective observational study with the ESS throughout the first 24 h postoperatively in 207 surgical in-patients. We compared ESS with Modified Early Warning Systems (MEWS), and postoperative journal information. We subsequently validated ESS by addressing recognized quality criteria for measurement of health status questionnaires.

**Results:**

A call-out value of ESS ≥10 correlated with MEWS > 0 values and journal information about postoperative concerns with a sensitivity of 94% and 92%, respectively. All serious safety issues were identified with the ESS ≥ 10, and a higher number of quality issues were identified than with routine care or MEWS. We obtained positive ratings for six out of seven tested criteria of questionnaire quality; one criterion had an indeterminate rating.

**Conclusion:**

ESS fulfils suggested criteria for score quality validation and reflects the patient’s postoperative status adequately and with high sensitivity. Further clinical trials are warranted to evaluate the usefulness of ESS as a simple tool for assessment of the postoperative safety and quality of patients.

**Electronic supplementary material:**

The online version of this article (doi:10.1186/s12871-017-0344-0) contains supplementary material, which is available to authorized users.

## Background

In modern perioperative care, safety is a primary concern, although in-hospital mortality in a large, mixed surgical French adult populations recently was reported to be as low as 0.5% [1]. Much more frequent, although less serious, are the problems of patient perceived quality, especially in the post-operative period.

In a survey of 2252 patients, 55% reported that they suffered from unsatisfactory pain treatment postoperatively [2]. Even so, a considerable number of patients experience adverse effects of the analgesic treatment given. A recent review including data from 183 studies comprising more than 100.000 patients undergoing postoperative pain treatment, showed a high incidence of side-effects from pain treatment: 25% suffered from nausea, 20% from vomiting, 15% from pruritus and 23% from urinary retention, whereas no cases of serious respiratory depression were reported. Notably, still 24% of the patients received too little analgesia for their pain, and only 2.6% were classified as receiving too much analgesics or sedatives [3]. Side effects are most typically opioid - induced. Thus, it may be argued that health care personnel should aim at better surveillance of peri-operative quality, both analgesic effects and side-effects.

A universal postoperative registering system for quick analysis of the overall safety, quality and efficacy by the same tool may create a higher level of confidence and security within the nursing staff. Moreover, lack of feedback on the analgesic effects or fear of side-effects of especially opioid administration, may lead to under dosing, whereas improper non-systematic monitoring of side-effects may lead to overdosing. Cases of fatal outcomes in hospital as well as at home after discharge has been described in this context [4–8].

As long as the patients remain in the post-anaesthesia care unit (PACU) with continuous monitoring and observation by highly trained staff, there are abundant tools and measures at hand to ensure both safety and quality of the treatment. However, after discharge to an ordinary ward, there are less staffing and monitoring resources, and the patients may even be left alone for shorter or longer periods of time.

Other scores available for postoperative assessment are usually designed for a specific purpose and not a global longitudinal evaluation. Examples include score for readiness for PACU discharge (e.g. the Aldrete score), score for the long-term outcome and patient satisfaction (e.g. PQRS) or score for acute medical deterioration (e.g. MEWS). We were looking for a clinical tool to address longitudinal quality and safety in combination, as a routine audit for every postoperative patient, with a special focus on the time period from PACU check-out to discharge readiness from hospital.

We hypothesized that medical personnel responsible for postoperative care and pain treatment, especially at the ward, but also in the PACU, may benefit of the feedback from a simpler, but still complete, scoring system. The system should survey safety aspects of changes over time in consciousness, respiratory and circulatory parameters as well as patients’ subjective postoperative perceived quality, including effect of pain treatment and side effects. The scoring system should include a call-out algorithm for the nursing staff to get help and guidance from the physician on call.

For this purpose, we started with an algorithm-based monitoring system with multiple simultaneously recorded variables, as a prototype tool for assessment of quality and safety of postoperative treatment [9, 10]. After a successful pilot, we decided to develop this tool further through a proper consensus, documentation and validation process. The goal of the present investigation and report is to present the results of this development and validation.

## Methods

The Regional Committee for Medical and Health Research Ethics South East evaluated this observational prospective study part of the validation process as a quality assessment exempt from informed consent of the patient (ref 2014/580 A). The patient protocol was approved by the Local Data Inspectorate of Vestre Viken Hospital Trust, Drammen, Norway (ref 2015/4793) who is the owner and administrator of Kongsberg Hospital.

We address four aspects of the validation process:

1. Based on the prototype score and a Delphi process [11] with a panel of recognised experts, we sought to obtain consensus on the contents of the score, which after revision was called the Efficacy Safety Score (ESS).
2. We validated ESS in a new group of patients encompassing independent scoring by two nurses.
3. Since no unified previous tool existed which could be compared with the whole score, we compared ESS with the modified Aldrete score [12] and the Modified Early Warning Score (MEWS) [13], which have a focus on the safety aspects of recovery.
4. Finally, we evaluated the ESS as to quality criteria proposed for the validation of health status questionnaires by Terwee and co-workers [14].

### Development of the score tool

Table 1 shows the different steps in the process of developing the postoperative score tool towards the ESS version, which then were validated. Initially, we searched the literature and found several postoperative assessment scores [12, 15–22]. Since we found no simple system constructed for global monitoring of the general status, as well as efficacy and side - effects of pain treatment after discharge from PACU, we developed a prototype score tool, “the Kongsberg satisfaction score”, (KSS) for this purpose. The prototype was based on information about consciousness, postoperative nausea/vomiting (PONV) and the degree of experienced pain at rest and during mobilization using Verbal Numeric Rating Scale (VNRS) [23]. We intended to make the score simple and easy to perform, facilitating the everyday use on all postoperative patients. This also includes baseline aspects related to regional anaesthesia, including neuraxial blocks. Still, in these situations there are some specific issues of nerve block characteristics and safety issues which are specific and different depending upon the type of regional anaesthesia. In order to maintain a simple, universal and quick to use all-purpose score, these specific aspects of different regional anaesthesia techniques are not included in our score, but should be added to the use of ESS on an individual case bases.

**Table 1:** Steps in the process of developing Efficacy –Safety Score (ESS)

Steps	Process
1.	Comprehensive literature review to identify current postoperative scales and scores to determine their limitations.
2.	Identification of aspects of interest regarding postoperative patients based on empirical experience.
3.	Pilot study (*n* = 182) with the prototype score to identify possibilities and pitfalls for a novel tool for postoperative use [9, 10].
4.	Identification through a Delphi-project the aspects considered relevant for clinicians to make a postoperative assessment [11]. Table 2.
5.	Refining the score and system after pilot study and Delphi-project.
6.	Writing of a protocol and conducting of a validation study in 207 patients.
7.	Validation of the ESS against the criteria set by Terwee et al. [14] Table 4.

The prototype score tool was tested on a pilot population of 182 patients, previously reported [9]. In parallel with testing the prototype score tool, we performed a modified Delphi-process with three iterations until consensus. An international group of ten recognized experts in postoperative care made suggestions about information needed in order to give an adequate and sufficient evaluation of the state of the clinical condition after discharge from PACU. Consensus was pre-defined as more than 80% agreement between the participants. The result of this process, shown in Table 2, was used to revise the prototype score from the pilot-study, as the issues of consciousness and general condition was added into the ESS for the subsequent validation study.

**Table 2:** Arrangement and results of Delphi-process

Questions in modified Delphi process	Answer given with consensus, >80% concordance, *n* = 10. (% agreement).
1. To make a judgment of a patient’s postoperative condition during the first 24 h after surgery, what clinical information do you need?	✓ Blood Pressure (100) ✓ Breathing Frequency (100) ✓ Pain (100) ✓ Pulse Frequency (100) ✓ Diuresis (90) ✓ Level of Consciousness (90) ✓ Nausea (90) ✓ Oxygen Saturation (80)
2. How will you assess and enter a patient’s pain postoperatively?	✓ Numeric Rating Scale/Visual Analogue Scale at rest (100) ✓ Numeric Rating Scale/Visual Analogue Scale during movement/coughing (100)
3. How will you assess and enter the degree of mobilization of a patient after surgery?	✓ Mobilized in bed/sitting in bed (100) ✓ Walk some steps with/without support (100)

Depending on the patient’s status or complaints, each of the clinical features in the final version were scored from 0 to 15 and summarized in a total score (Table 3). From the pilot study it was suggested that an ESS score ≥10 was an appropriate cut-off value for serious problems in need of immediate consultation with a doctor or other expert health personnel. The project group discussed at which single events (such as respiratory or cardiovascular problems) or combination of minor events a clinical intervention was needed. It came out that single serious events or combination of minor events with score of more than 10 seemed to be a reasonable cut-off point to test. However, the study was designed to re-consider retrospectively the cut-off point in case of some serious events were missed by this cut-off point (i.e. cut off point should be lower), or too many false calls for intervention was initiated (i.e. cut off point should be higher). For this reason, the cut-off value of ≥10 was tested prospectively, allowing for a post-hoc evaluation of the proper cut-off value.

**Table 3:** Description of Efficacy Safety Score (ESS) as revised after pilot-study and Delphi-process

Mental status	Score
Awake and alert patient	0
Awake patient, but influenced by drugs. Difficulties in communication.	5
Acutely confused, upset/uneasy, hallucinated or euphoric patient	10
Unresponsive patient	15^*^
Postoperative nausea and vomiting (PONV) status	
No postoperative nausea or vomiting	0
Postoperative nausea only	5
Postoperative nausea and vomiting/retching	10
Pain status at rest	
No postoperative pain	0
Low intensity postoperative pain (VNRS 1–3)	1–3
Moderate intensity postoperative pain (VNRS 4–6)	4–6
Severe intensity postoperative pain (VNRS 7–10)	7–10
Pain status during movement	
No postoperative pain	0
Low intensity postoperative pain (VNRS 1–3)	1–3
Moderate intensity postoperative pain (VNRS 4–6)	4–6
Severe intensity postoperative pain (VNRS 7–10)	7–10
General condition status	
No remarks	0
Minor discomfort (e.g. light-headedness, minor itching, blurred vision, decreased urination etc.)	5
Excessive discomfort (e.g. severe dizziness, itching, restlessness, urine retention, sensation of cold/warmth, cold sweating)	10
Acute circulatory abnormalities (blood pressure ≤80 or ≥200 mmHg, heart rate ≤40 or > 110)	15^*^
Acute respiratory abnormalities (dyspnoea, respiration rate < 9 or >20/min, long pauses in breathing, shallow breathing)	15^*^

^*^Any single score of 15 (on either consciousness, circulation or respiration) should call for IMMEDIATE activation of acute assistance with the patient

### Validation study

A prospective observational study was conducted at Kongsberg Hospital to monitor postoperative in-patient status with the ESS during the first postoperative 24 h to provide data to validate the ESS score against published general criteria for validation of verbal scores set out by Terwee et al. [14] (ref. Table 4). Data were collected between March and August 2015 at the Departments of Anaesthesiology, High dependency Unit, General Surgery, Orthopaedics, Obstetrics and Gynaecology, and Ear-, Nose- and Throat at Kongsberg Hospital, Kongsberg, Norway.

**Table 4:** Terwee et al. quality for health status questionnaires

1. Content validity	The extent to which the concepts of interest are comprehensively represented by the items in the questionnaire
2. Internal consistency	The extent to which items in a (sub)scale are inter-correlated, thus measuring the same construct
3. Criterion validity	The extent to which scores on a particular questionnaire relate to a gold standard
4. Construct validity	The extent to which scores on a particular questionnaire relate to other measures in a manner that is consistent with theoretically derived hypothesis concerning the concepts that are being measured.
5. Reproducibility	a. Agreement The extent to which the scores on repeated measures are close to each other (absolute measurement error) b. Reliability The extent to which patients can be distinguished from another, despite measurement errors (relative measurement error)
6. Responsiveness	The ability of a questionnaire to detect clinically important changes over time.
7. Floor and ceiling effects	The number of respondents who achieved the lowest or highest possible score.
8. Interpretability	The degree to which one can assign qualitative meaning to quantitative scores.

- Prospective clinical study:

ESS was used by the regular staff at the ward after education was given. The registration chart was on paper sheets and filled in by nursing staff hourly the first 8 h postoperatively (also including the PACU period), and then every 4 h for the next 16 h at the ward. The highest score in each time period of 1 h in the PACU and 4 h at the ward was registered.

The inclusion criteria were all operated patients who were expected to be treated and observed in hospital overnight. Exclusion criteria were patients < 18 years of age or patients with poor communication capabilities.

b) Quality assurance based on individual patient data:

In parallel, we collected several parameters from the complete journal of the patient (Table 5), in order to check if important information from the regular routine monitoring and registration was missed in the ESS registration of the individual patients. From the PACU stay we checked the charts of regular PACU monitoring on vital signs and medication, as well as the fulfilment of PACU discharge criteria form by the modified Aldrete score [12]. At the ward, we used the Modified Early Warning Systems (MEWS) [13], applied on the scheduled nursing notes to validate the ESS. The MEWS is a validated tool developed to identify patients with acute deterioration, including four physiological measurements (i.e. systolic blood pressure, heart rate, respiratory rate, temperature) and one observation (level of consciousness), with a total score from 0 to 14.

**Table 5:** Information extracted from cohort medical journals

1. Information about pain, nausea and vomiting the first 24 postoperative hours
2. Information about medication given the first 24 postoperative hours
3. Time to readiness for discharge from recovery unit (Modified Aldrete Score ≥9)
4. Not-scheduled contacts/visits by physician
5. Re-admittance to high dependence unit or re-operation
6. Result of performed Modified Early Warning Score at the ward
7. Time spent at recovery unit, total hospital stay and 30-days mortality

c) Validation of the ESS score (see criteria in Table 4 and Additional file 1: Addendum 1):

For formal validation of the ESS we used “The Quality for Health Status Questionnaires criteria” listed by Terwee et al. [14] as a guide for the process. These criteria are explicit requirements for formal validation of questionnaires. The detailed descriptions of the criteria are given in Additional file 1: Addendum 1.

### Statistical methods

All collected data were registered in Microsoft® Excel® 2010 for PC, version 12.0. Patients were ASA classified and sorted in groups according to the type of surgery and anaesthesia. The results are given as numbers and percentages for selected groups, and as means ± standard deviations (SD) for age, weight, height and Body Mass Index (BMI). Statistical analyses were conducted using IBM® SPSS® Statistics Version 21 for one-way Analysis of Variance (ANOVA), estimating Intra-class Correlation Coefficient (ICC) and post-hoc Bonferroni correction. For between-group differences when the data were compared by gender and ASA-score, we used repeated-measures ANOVA.

## Results

We enrolled 207 patients scheduled for inpatient surgery for validation of the ESS. Two patients were excluded due to age under 18. The demographic details for the cohort are shown in Table 6.

**Table 6:** Demographics and operative variables in cohort, *n* = 207

	Range	Mean (±Standard Deviation (SD))
Age, yr	18–92	57,9 (±16,4)
Height, m	1,50 – 1,98	1,69 (±0,09)
Weight, kg	40–160	77 (±15,7)
Body Mass Index (BMI)	17,2–37,6	27,0 (±4,9)
Duration of anaesthesia, min	29–210	107 (±57)
	*n*	Percent
Gender, female	165	79,7%
American Society of Anesthesiologists Status:		
ASA I	71	34,3%
ASA II	112	54,1%
ASA III	22	10,6%
ASA IV	2	1,0%
Planned Surgery	178	86,0%
Type of surgery		
Orthopaedic - total Knee/total hip joint replacement Fracture fixation Other	99 28 / 50 19 2	47,8% 13,5% / 24,2% 9,2% 1,0%
Gynaecological - total Vaginal/open hysterectomy Laparascopic hysterectomy Vaginal repair surgery Caesarean section Other	94 37 / 7 22 16 8 4	45,4% 17,9% / 3,4% 10,6% 7,7% 3,9% 1,9%
Ear, nose and throat - tonsillectomy	14	6,8%
Type of anaesthesia		
“Target”-control infusion(TCI) of propofol and remifentanil	94	45,4%
Spinal anaesthesia(SA)/Epidural anaesthesia(EDA) ± sedation(sed)	73 / 2	35,2% / 1,0%
TCI + EDA/Regional block(RB)/SA	28 / 2 / 2	13,5% / 1,0% / 1,0%
Gas anaesthesia(GA): sevoflurane and fentanyl	6	2,9%

Evaluation of measurement properties criteria:

### Content validity

After the modified Delphi-process, in which we regarded agreement between the experts of 80% or more as consensus, we adjusted the prototype score into the ESS, for subsequent validation study (Table 3).

The ESS is built up from already clinically validated domains: Four level judgement of mental status, 11-point numerical pain assessment and MEWS in general status. Digitalized expressions from these domains are incorporated into one simple tool together with clinical assessments of subjective comfort/discomfort for the postoperative patient. These individual domains are all related but independent factors to the clinical status of patients in the postoperative phase, see Table 7.

**Table 7:** Mean values (SD) of Efficacy Safety Score (ESS) and mean values of individual domains deconstructed, *n* = 207

Postoperative hours	1	2	3	4	5	6	7	8	12	16	20	24
ESS	5.5 (7.4)	3.9 (4.6)	3.7 (4.4)	3.6 (4.5)	3.8 (4.5)	3.7 (4.3)	4.0 (4.2)	4.2 (4.2)	4.6 (5.8)	3.7 (4.5)	3.9 (4.4)	3.7 (4.0)
Mental status	0.5 (2.0)	0.1 (0.9)	0.1 (0.9)	0.0 (0.4)	0.0 (0.4)	0.0 (0.0)	0.0 (0.0)	0.0 (0.0)	0.1 (0.3)	0.0 (0.0)	0.0 (0.0)	0.1 (1.0)
PONV	0.4 (1.5)	0.5 (1.7)	0.6 (2.0)	0.3 (1.5)	0.4 (1.6)	0.5 (1.8)	0.6 (1.9)	0.6 (2.0)	0.,5 (1.8)	0.4 (1.5)	0.5 (1.8)	0.3 (1.4)
Pain status at rest	2.0 (2.8)	1.5 (2.0)	1.3 (1.6)	1.4 (1.6)	1.4 (1.7)	1.3 (1.6)	1.4 (1.5)	1.5 (1.8)	1.6 (1.8)	1.4 (1.8)	1.4 (1.5)	1.1 (1.2)
Pain status during movement	2.0 (2.8)	1.6 (2.1)	1.5 (1.8)	1.7 (1.8)	1.7 (1.8)	1.7 (1.8)	1.8 (1.7)	2.0 (2.0)	2.0 (2.1)	1.8 (2.1)	1.9 (1.9)	1.9 (1.9)
General status	0.6 (2.1)	0.2 (1.3)	0.2 (1.0)	0.2 (1.3)	0.3 (1.7)	0.2 (1.4)	0.2 (0.9)	0.1 (0.7)	0.3 (1.7)	0.2 (0.9)	0.2 (0.9)	0.3 (1.7)

### Internal consistency

Not relevant for ESS (see methods).

### Criteria validity

#### ESS and discharge from PACU checkout

The PACU discharge criteria used in the study hospital were the modified Aldrete score ≥9 [12] in addition to registration of none or mild pain (VNRS ≤ 3) and absence of postoperative emetic symptoms. The study hospital routinely recorded the time of complete achievement of all criteria, without specific notes on the individual criteria. The mean ESS at the time of fulfilling these criteria was 2.84 (SD 2.82).

#### ESS versus MEWS

In the 16 patients (8%) with a positive MEWS score (1 to 5), we found a correlation with ESS ≥ 10 for 15 patients (sensitivity against MEWS = 0.938). The one patient with positive MEWS and ESS < 10 presented with tachypnea (rate 20/min), without clinical implications. There were no patients with a positive MEWS in the remaining 106 patients (51% of sample) with ESS ≥ 10 (specificity against MEWS of 0.445).

#### ESS versus patient journal information

A total number of 121 patients (58%) had an ESS score of ≥10, as an indication of a relevant safety or quality problem. A total of 99 patients had a note in their journal about postoperative concerns; such as pain, nausea, vomiting, circulation irregularities, respiration remarks and/or notes on general condition. Ninety-one of these patients had an ESS ≥10. Eight patients had ESS < 10 where of 6 with minor issues not being significant safety concerns; two patients had episodes of nausea, one of anxiety, two of moderate “dull” pain and one of disorientation. Two potential safety issues were missed by the ESS, probably due to missing ESS registrations for the relevant period of their recovery: one had bradycardia (42beats/min) and one had tachycardia (115beats/min). Of the remaining 91 patients with a postoperative note on either safety or quality issues in their journals and concomitant ESS ≥10, we found three patients with syncopation -, one with apnoeic periods after opioid administration -, four with tachycardia >129/min, two with systolic blood pressure <90 mmHg and two in need of extra oxygen supply because of SaO_2_ < 90%. For the remaining 79 patients there were relevant quality issues, but no aspect of safety problems involved.

The overall sensitivity for this comparison of ESS ≥10 against relevant journal information was 0.919 and the specificity was 0.722.

### Construct validity and responsiveness

For the sub-group having total joint replacement (*n* = 78) the mean ESS for the first two hours was 1.5 (±2.4) and 8.7 (±6.6) for regional anaesthesia and general anaesthesia, respectively. Estimated mean difference was −7.2 (95% Confidence intervals (CI) 5.17–9.23, *P* < 0.0001) in significant favour of regional anaesthesia.

For the gynaecological hysterectomy sub-group (*n* = 66), the mean ESS for the first 2 h was 4.7 (±4.4) and 7.4 (±6.2) for regional anaesthesia and general anaesthesia, respectively. Estimated mean difference was −2.7 (95% CI 0.04–5.36, *P* = 0.0466) in significant favour of regional anaesthesia for this sub-group.

This confirms the ability of the ESS to significantly differentiate two groups of expected different recovery quality in two different subpopulations. Looking at individual differentiation is not clinically relevant as the patients with best recovery safety and quality in the general anaesthesia group will be expected to overlap with the patients with most recovery problems in the regional anaesthesia group.

### Reproducibility

#### Reliability

The estimated ICC from the reliability testing was 0.953. This value is larger than 0.70, which was suggested for positive rating by Terwee et al. [14].

#### Agreement

The estimated SEMagreement is 0.197 (SEMagreement = SD x (√1-ICC)). Estimated SDC is 1.26 (SDC = 1.96x √2x SEMagreement). This SDC is less than the defined MIC of 1.30 and the statistically estimated MIC of 2.33, as described under interpretability, which gives a positive rating according to Terwee et al. [14].

### Floor and ceiling effects

The theoretically possible highest ESS is 60, and no patient in the cohort approached this value. Floor effect, ESS = 0, which is the patient’s habitual situation of 100% well-being, was found in 4.8% (10 patients) for the whole observation period of 24 h.

### Interpretability

**Fig. 1:**
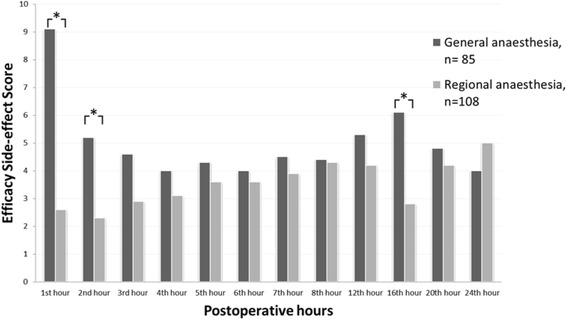
Mean values of Efficacy Safety Score (ESS) for subgroups of regional versus general anaesthesia during the first 24 postoperative hours. *P=0.05

The standard deviation of the ESS for the cohort at all time points was 4.65. Correspondingly MIC is 2.33, estimated by the distribution method. Two significant subgroup effects were identified: this involved the variables age and type of anaesthesia. Bonferroni post hoc testing showed the effect to be located between the patients aged ≤65 or ≥75 years, respectively, at the 1st and 24th hour postoperatively. Effect was also shown to be located between the general anaesthesia versus the regional anaesthesia groups at the 1st, 2nd and 16th postoperative hours, Fig. 1. We found no significant between-group differences when the data were compared by gender and ASA-score.

## Discussion

In this study on the validation of our newly developed ESS score, we found the score sensitive and useful in routine postoperative care. Our pre-set call-out value greater or equal than 10 for calling for enforced attendance and help, seemed to be appropriate for further testing as no cases of serious events were missed by this call-out value. Although 51% of all patients qualified for the need of physician attendance by this call-out value, we think it is important to be on the safe side. It is better with some calls not being needed, than missing one urgent and important case. Also, in 88% of the call-outs there was a significant quality problem of patient care which should justify the involvement for the physician on duty.

With the ESS we addressed and fulfilled the seven relevant out of total eight validation criteria recently suggested by Terwee et al. [14]. Positive rating was assigned for six of the criteria, and an indeterminate rating was assigned for the final criterion of validity due to the lack of convincing arguments that the suggested gold standards are definite as such.

When developing health-status questionnaires, no empirical evidence exists concerning the final choice of quality criteria. The criteria suggested by Terwee et al. [14] are based on those of the Scientific Advisory Committee of the Medical Outcomes Trust [24]. Still, the Terwee et al. [14] checklist is not itself a gold standard to determine quality of a questionnaire, and the checklist does not determine which is the best questionnaire in an everyday clinical situation. The checklist is however recognized as a tool, which provides a systematic review of measurement properties to rate the quality of a questionnaire or test-battery, and considered helpful when introducing a new scoring system.

It is important that the use of the new scoring system never result in any delay in emergent situations when patient’s safety is acutely threatened. For this reason, any one of the three observations on: unconsciousness, acute circulatory problems or acute respiratory problems is designated a score of 15, which should call for immediate actions as to get qualified help.

For the quality part of the ESS related to pain and nausea/vomiting/retching, the scaling is based on the well-established verbal 11-point numerical scale (0–10) used for assessment of pain [23]. Postoperative pain is a major concern for patients after surgery, and this is reflected in the score by evaluating pain both at rest and at movement, also because mobilization is an essential aspect of recovery after surgery. We regarded unconsciousness as a condition being more concerning from a safety point of view than severe pain and vomiting/retching, and gave consciousness a score range from 0 to 15. The same considerations were made for circulatory and respiratory abnormalities.

The empirical weighting of the different aspects of the score may be questioned, but the purpose of the score is to give the nursing staff a quick and simple tool in analysing the patient’s postoperative status. It does not include all aspects of postoperative care, and is not designed to explore in detail the degree of severity of the patient’s condition. It consists of specific aspects of recovery problems summarized to a single number as a call-out algorithm. It is made to identify patients who need their care adjusted in a busy daily care. The goal is to make a simple judgement of every patient with specific questions on clinical concerns important for the postoperative patient. When performed in daily clinical situations, the score is deconstructed into these individual domains and the staff can make clinical decisions from this. We have data from patients having high ESS due to severe postoperative pain, which continues having high ESS after the pain is treated, but now side-effects like retching and vomiting is the cause. This balanced clinical approach to the patients with explicit questions about all the domains is what contributes to ESS as a clinical tool, where summarized input from all the domains reflects the clinical situation. Retrospective extraction of individual domain data helps to analyse postoperative care retrospectively, and may improve quality of care.

We consider the low specificity under criteria of validity with comparison to MEWS to be due to the different aspects and aims of the two scores. The ESS reflects the quality and safety of the treatment given postoperatively in a wider scope and with a sensitive scaling, whereas the MEWS is constructed for safety issues primarily, with a simple dichotomous “no problem” versus “serious problem” score.

As to comparison of ESS with information in the medical journal, the latter is often sporadically and non-methodologically written. Still, all serious problems will be reported in the journal, either as such or as notes of the doctor being called upon, or extra drugs being given. The findings of very few minor clinical complaints documented in the medical journal, may explain the sub-optimal specificity for this comparison.

The ESS scores peaked at 16 h registration for general anaesthesia, whereas the opposite (i.e. low values) were shown for regional anaesthesia. This may have to do with the resolution of regional blocks at this time for many patients, with subsequent pain and administration of systemic analgesics with some side-effects. In the general anaesthesia group, it may have to do with the morning round at the ward, topping up analgesic treatment.

In a recent review, Bowyer and co-workers identified 11 different scoring tools of recovery status, checking and documentation. Out of those the authors concluded that the Postoperative Quality of Recovery Scale (PQRS) was best in assessing recovery in all relevant domains, including physiological, nociceptive, emotive, activities of daily living, cognition and patient satisfaction [25]. It addresses recovery over time and compares individual patient resumption of capacities data with base line, and is an acceptable and appropriately validated method for identification of individual patient recovery. However, as the PQRS is designed with 22 questions to cover all aspects of the full post-operative course, from leaving the operating room until full resumption of normal activities, it is perceived as cumbersome and time consuming to use. Further, it necessitates a baseline registration in order to make proper value to the dichotomous outcome of either worse or similar/better as compared with the pre-operative status. For these reasons, even the authors suggest PQRS basically as a tool for research, not for everyday clinical practice [15].

Other scoring systems are also available and extensively used, such as the MEWS system [13] and the Aldrete score [12]. The MEWS system is a simple system on safety issues only, and does not take into account quality aspects. This was also evident in our test of the MEWS versus ESS in the present study.

The Aldrete score is specially designed as a dichotomous “yes” or “no” tool for PACU discharge, with no grading of safety and quality issues. Further, it does not take into account the longitudinal progression of post-operative status with time.

After the prospective study an evaluation report was written by the nurses about ESS. The report described improved communication and it emphasized that the call-out algorithm made it easier to get immediate help and assistance for postoperative patients.

### Limitations of the study

It is a limitation of this study that the observation period for ESS only was for the first 24 postoperative hours. This was chosen in order to focus on safety problems, which are more frequent during the first 0–24 h, for the important sensitivity of the scoring system on this aspect. A further limitation is that data described in this paper reflects the cohorts and surgical case load in the hospital studied. There was a bias towards many female and elderly patients going through planned orthopaedic surgery. It may also be a weakness of the study that most of the surgery performed was planned, and that most patients had ASA status 1 or 2. While obviously the ESS will change upon efficient treatment of e.g. overt nausea or strong pain, this aspect was chosen to not be included in this first report of the score for sensitivity and specificity. A single study like this is not sufficient to claim complete clinical validation for the ESS, but it is useful for evaluating feasibility, and to check whether the score detects what it is supposed to.

## Conclusions

ESS is constructed to give the nursing staff sufficient clinical relevant information about postoperative patient status and thereby a possibility to improve patient safety and enhance quality. Also, to provide a call-out algorithm (i.e. ESS >/= 10) for immediate call for competent guidance and help. ESS is a simple scoring system to apply, routinely conducted in less than one minute, making it a useful tool for the staff in regular everyday situations. It is based on clinically information that is easy accessible, and minimal training is needed prior to use. The findings from this validation project indicate that ESS may contribute positively to the field of postoperative management. ESS fulfils suggested criteria for score quality validation and reflects the patient’s postoperative status adequately and with high sensitivity. However, in order to evaluate the usefulness of ESS in everyday practice and beyond 24 h hospital stay, further clinical trials are needed. The next step will be to test the sensitivity and specificity of the ESS in large patient populations, including seriously ill, patients, emergencies and major surgical cases. Also, to test if use of the score may result in better patient safety and satisfaction, as well as being perceived as useful in an everyday setting by the clinical staff.

## Additional file

Additional file 1: A detailed description of the criteria we used for as a guide for formal validation of the ESS, “The Quality for Health Status Questionnaires criteria” listed by Terwee et al. [14], is presented in Addendum 1. (DOCX 27 kb)

## Abbreviations

ANOVA: Analysis of variance
ASA: American Society of Anesthesiologists
BMI: Body mass index
CI: Confidence intervals
EDA: Epidural anaesthesia
ESS: Efficacy safety score
GA: Gas anaesthesia
ICC: Intra-class correlation coefficient
MEWS: Modified early warning score
MIC: Minimal important change
PACU: Post-anaesthesia care unit
PONV: Postoperative nausea and vomiting
PQRS: Postoperative quality of recovery scale
SA: Spinal anaesthesia
SD: Standard deviation
SDC: Smallest detectable change
SEM: Standard error of measurement
TCI: Target-control infusion
VNRS: Verbal numeric rating scale
