# Supplementary material for: Development and validation of the Efficacy Safety Score (ESS), a novel tool for postoperative patient management
Source: BMC Anesthesiol. 2017 Mar 28;17:50. doi: 10.1186/s12871-017-0344-0 (PMC5371277; doi:10.1186/s12871-017-0344-0)
Supplement: Additional file 1: — A detailed description of the criteria we used for as a guide for formal validation of the ESS, “The Quality for Health Status Questionnaires criteria” listed by Terwee et al. [14], is presented in Addendum 1. (DOCX 27 kb) [file 12871_2017_344_MOESM1_ESM.docx]

**Additional file 1: ADDENDUM 1**

We evaluated the below criteria according to Terwee et al [14].

**Content validity**

This is the ability of the score to contain and adequately ask for the relevant issues, leaving out issues, which are non-relevant for the purpose. Content validity was established by our two-step process: First, the pilot-study [9, 10] with the prototype score was based on empirical clinical experience, comprehensive literature review and a thorough evaluation of common side-effects of anaesthesia and pain treatment (table 1). Thereafter we established a Delphi-project to collect expert opinions for the topic, and incorporated this in the novel tool for postoperative assessment, the ESS. Consensus in this Delphi-project, with three iterations to ten international experts, was defined as an agreement of 80% or more.

**Internal consistency**

This is the issue on having a score without internal contradictions on identical issues. Given that the ESS is a score based on different aspects of rather complex clinical phenomena that do not have to be correlated, the criterion of internal consistency is not relevant [26, 27] or possible to test.

**Criterion validity**

This is whether the criteria for acceptable versus non-acceptable values of the questionnaire items fit with previously recognized, validated and accepted standards. We undertook three measures for this purpose. There is no validated “gold standard” for the global postoperative patient status of the whole 0-24 hrs period after surgery. Criteria used for discharge from the recovery unit to the general ward, for instance, the modified Aldrete score [12, 16], is not formally validated in the literature, but has been used for validation purpose of the ESS. The Modified Early Warning Score (MEWS) is validated [13], but do only address serious events and patient safety, and not so much the perceived quality for the patient. We examined to which degree the scores of the ESS corresponded to MEWS. In order not to miss any events, we also examined the journals of the 207 patients to search for answers to the questions in table 5.

**Construct Validity and Responsiveness**

This has to do with whether the output of the tested score corresponds to reported end results, both in terms of reflecting only relevant issues (specificity or validity), and not missing cases with relevant problems (sensitivity or responsiveness). A positive rating on construct validity and responsiveness is given where specific hypotheses are formulated and at least 75% of the results are in accordance with these hypothesis [14]. For this purpose, a priori hypotheses were generated and tested regarding the relationship between the ESS and clinical outcome. From our own experience and data on nausea and pain in the literature [28], we hypothesized a lower initial ESS for patients receiving regional anaesthesia compared to patients not having regional anaesthesia for the same type of surgery. Patients who underwent total joint replacement and hysterectomy were relevant sub-groups to study for this aspect. If the ESS could confirm this hypothesis, this would be a sign of validity. To distinguish clinically important changes from measurement error, we checked if the minimal important change (MIC) was larger than the smallest detectable change (SDC) in a relevant subgroup of pain registrations. MIC was defined by using well documented results where a reduction in pain severity equivalent of mean 1.3 points or more on a Numeric Rating Scale (NRS) was clinically significant [29]. The NRS scale we used was designed to detect 1.0 as SDC. We also estimated the MIC by using the conservative distribution method, saying that MIC is at least 0.5 x Standard Deviation [30].

**Reproducibility**

This has to do with the test or questionnaire producing the same results when tested repeatedly on a subject in the same situation. The postoperative situation is dynamic and thereby longitudinal reproducibility is, by nature, difficult to make. However, a random investigation of 54 patients of the total sample was undertaken with the purpose of testing reliability. Two regular staff members simultaneously and independently noticed ESS during the first hours. The results were blinded for the other staff members.

**Reproducibility: Reliability**

Reliability has to do with the ability of the score to record different results in different patients with different status. Reliability can be measured by estimating the Intraclass Correlation Coefficient (ICC). The model chosen in SPSS is ICCagreement: Two-way random, single measure - ICC. A positive rating is given for reliability when the ICC is at least 0.70 in a sample of at least 50 patients [14].

**Reproducibility: Agreement**

Agreement has to do with how close the results of the repeated measurements are, by estimating the measurement error in repeated measurements. The measurement error can be expressed as the standard error of measurement (SEM) [31]. SEM agreement equals the square root of the error variance of an ANOVA analysis including the systematic difference: SEMagreement =SD x (√1-ICC). This was then converted into the Smallest Detectable Change (SDC) using the formula SDC=1.96 X √2 X SEMagreement [32]. A positive rating for agreement is given when SDC is smaller than the defined Minimal Important Change (MIC) [14].

**Floor and ceiling effects**

If too many patients either have the minimum or maximum score, this might be an indication on the scale of the score not being adequate for the range of outcomes studied. Still, with quality scores it may be fully acceptable to have a high number of patients with the maximal score for quality (i.e. ESS=0), whereas a high number on score for minimal quality will warrant further expansion of the scale. Floor and ceiling effects were defined as more than 15% of patients having lowest or highest possible ESS, respectively. For this purpose, the score frequencies were examined separately for the whole period of 24 hours. A positive rating is given for absence of floor and ceiling effects in at least 25% of the patients [14].

**Interpretability**

The definition of interpretability is the degree to which one can assign qualitative meaning to quantitative scores [33]. For this purpose, the subgroup analyses planned a priori were examination of ASA-score, gender, age and type of anaesthesia (general anaesthesia versus regional anaesthesia). Ear, nose and throat surgery was excluded from the type of anaesthesia subgroup. A positive rating is given when mean and SD scores are presented of at least four sub-groups, and MIC is defined [14].

26. Fayers, P.M. and D.J. Hand, *Causal variables, indicator variables and measurement scales: an example from quality of life.* Journal of the Royal Statistical Society: Series A (Statistics in Society), 2002. **165**(2): p. 233-253.

27. Streiner, D.L., *Being inconsistent about consistency: when coefficient alpha does and doesn't matter.* J Pers Assess, 2003. **80**(3): p. 217-22.

28. Kettner, S.C., H. Willschke, and P. Marhofer, *Does regional anaesthesia really improve outcome?* Br J Anaesth, 2011. **107 Suppl 1**: p. i90-5.

29. Todd, K.H. and J.P. Funk, *The minimum clinically important difference in physician-assigned visual analog pain scores.* Acad Emerg Med, 1996. **3**(2): p. 142-6.

30. Norman, G.R., J.A. Sloan, and K.W. Wyrwich, *Interpretation of changes in health-related quality of life: the remarkable universality of half a standard deviation.* Med Care, 2003. **41**(5): p. 582-92.

31. Stratford, P., *Reliability: consistency or differentiating among subjects?* Physical Therapy, 1989. **69**(4): p. 299-300.

32. Beckerman, H., et al., *Smallest real difference, a link between reproducibility and responsiveness.* Qual Life Res, 2001. **10**(7): p. 571-8.

33. Lohr, K.N., et al., *Evaluating quality-of-life and health status instruments: development of scientific review criteria.* Clin Ther, 1996. **18**(5): p. 979-92.
